# ‘Cut medicine for me’: addressing suboptimal dosing of antimicrobials as a critical issue to combat AMR in Nigeria

**DOI:** 10.1093/jacamr/dlae131

**Published:** 2024-08-09

**Authors:** Kenneth Chukwuebuka Egwu, Maryam Abdulkarim, Shadrach Chinecherem Eze, Oluchi Mbamalu

**Affiliations:** Department of Pharmacy, University of Nigeria Teaching Hospital, Ituku-Ozalla, Enugu State, Nigeria; Department of Molecular Biology and Biotechnology, University of Sheffield, Sheffield, UK; Department of Pharmacy, Federal Teaching Hospital, Ido-Ekiti, Ekiti State, Nigeria; Department of Global Health, Faculty of Medicine and Health Sciences, Stellenbosch University, Cape Town, South Africa; Global Surgery Division, Faculty of Health Sciences, University of Cape Town, Cape Town, South Africa

## Abstract

Antimicrobial resistance (AMR) is a critical health challenge in Nigeria as in many other countries in the sub-Saharan region of Africa. Our article describes how the challenges in the regulation and operations of Patent and Proprietary Medicine Vendors (PPMVs) in Nigeria provide a blind spot for the underuse of antimicrobials. This article also sheds light on how patients’ antibiotic use and seeking behaviour facilitate this unwholesome practice. In addition, our article looks at the social determinants of this practice, such as poverty and poor education, and proffers solutions towards solving it. While previous research has investigated the knowledge, perceptions and attitudes of PPMVs towards antimicrobial use and AMR, our article is the first to critically raise concerns about the common practice of antimicrobial underdosing in Nigeria.

In sub-Saharan Africa, healthcare is provided mostly in the private sector, with out-of-pocket costs in Nigeria estimated at 76.2%.^1^ Within the informal private sector, Patent and Proprietary Medicine Vendors (PPMVs) are major providers of basic health, especially in the rural areas, where they treat between 15% and 83% of common childhood illnesses and other ailments.^2^ PPMVs are individuals who sell medicines on a retail basis for profit, without formal training in pharmaceutical sciences. To become a PPMV, one must be at least 21 years of age, be able to read and write in English, have good character as attested by two referees, and hold a secondary school-leaving certificate or a Pharmacy Technician Certificate from a Pharmacy Council of Nigeria (PCN)-accredited institution.^3^ Advocates of PPMVs have cited wider rural reach, longer operating hours and more consistent medicine availability as benefits; however, concerns about adherence to regulations, poor knowledge of medicines and quality of service have been noted.^1^

In Nigeria, PPMVs are subject to regulations of the PCN,^4^ which grants them permission to sell specific over-the-counter medicines (OTCs) while barring antimicrobial prescribing and dispensing (with the exception of dispersible amoxicillin tablets 125 mg), as well as any form of invasive procedure. Such restrictions are meant to promote safe use of medicines (including antimicrobials). However, in order to meet the demands of their customers, PPMVs have extended beyond their scope, engaging in unlicensed sales of all kinds of antimicrobials, especially antibiotics and antimalarial drugs, which are usually in high demand. Studies have identified their sales of antibiotics such as metronidazole, amoxicillin/clavulanic acid, cefalexin and ceftriaxone, antimalarial drugs such as artemether lumefantrine, dihydroartemisinin piperaquine, artesunate amodiaquine, chloroquine, halofantrine, quinine and sulfadoxine pyrimethamine.^5^ Poor knowledge of medicines among PPMVs has been reported to drive irrational antimicrobial use, which can predispose to antimicrobial resistance (AMR).^4^ However, when PPMVs received training on antimicrobial use and resistance, the sale of antibiotics without prescriptions decreased significantly, with untrained PPMVs twice as likely to sell antibiotics without a prescription compared with those who received such training.^6^

The major role of PPMVs appears to be one of salespersons meeting their customers’ needs.^5^ In striving to cater for the communities they serve as well as drive business, PPMVs are open to rationing medicine dosage, according to their patient’s pocket. ‘Cut medicine for me’ is a commonly understood request, and describes a situation where medicine dosages are rationed according to the patient’s financial capacity, often resulting in dispensing of suboptimal doses of antimicrobials. While the drive to make profit without knowledge or consideration of antimicrobial stewardship principles is of concern, such practices have also been linked to pressure from patients, especially those with low purchasing power.

Another practice involves bit-by-bit assemblage of different antibiotic classes to treat a suspected infection. Bit-by-bit assemblage of drugs in this context involves inappropriate combination of a few tablets of the same or different drug classes in divided doses for use by the patient. These drugs, often including antimicrobials, are mixed indiscriminately, with little or no regard for possible drug–drug interactions or AMR risk. Data on the prevalence of these practices are lacking; however, numerous studies have raised concerns about PPMVs’ handling of antimicrobials.^6,7^

Against the background of non-universal health coverage, limited and largely inaccessible options for treatment, patients are caught between cost and cure. Completing a full course of antimicrobial therapy can be an unaffordable luxury to many. Antimicrobial underdosing points to larger failure of health systems and a desperate attempt to stretch inadequate resources as far as possible. This underdosing can occur in two main forms: not completing the prescribed course of antibiotics; and providing a lower dose than recommended. With limited awareness of AMR drivers,^8^ patients can be led to believe or assume that fewer tablets will suffice, especially when their symptoms abate. Additionally, due to cost constraint, patients may receive a lower dose of antibiotics, such as 250 mg instead of a prescribed 500 mg or half a blister pack instead of the complete pack, which would be insufficient to fully eliminate the infection. This is a recurrent problem and is pervasive across the nation. In addition, patients’ and PPMVs’ limited awareness serve as fertile ground for underdosing, and creates blind spots, which promote inappropriate antimicrobial use without recognition of its detrimental effects. The pressure to satisfy immediate needs in a resource-constrained space, compounded by their own need to make business profits, can overshadow long-term consequences related to AMR.^9^ In such a context, the emergence of resistance and failure of subsequent therapies generally results in referral, albeit delayed, to formal healthcare facilities for life-threatening conditions, where additional antimicrobial therapies are likely to fail.^6^

Nigeria’s high AMR burden, with over 263 000 associated deaths, highlights a need for more prudent antimicrobial use.^10^ Research-identified gaps highlighted AMR education and training needs among PPMVs as well as the public; for instance, as it relates to the latter’s demand for antimicrobials.^6^

## Recommendations

The underuse and misuse of antimicrobials in Nigeria has significantly contributed to the alarming rise in AMR. A comprehensive strategy is crucial to combat this growing threat.

### Recommendation 1

Underdosage of antimicrobials by PPMVs emphasizes the need for adequate policy review. Above all, the criteria for being a PPMV should be raised. Applicants should have some level of knowledge of medicines.

### Recommendation 2

To enhance the purchasing power of the population, the government must prioritize expanding the National Health Insurance scheme to rural areas and reducing healthcare costs, thereby ensuring accessible and affordable healthcare for all. This should be complemented with sensitization on the implications of incomplete dosage of antimicrobials to promote behaviour change pertaining to antibiotic seeking and use. This can be achieved through public sensitization campaigns, incorporation of AMR on radio and TV programmes for wider reach, and engagement of PPMVs as agents of change.

### Recommendation 3

There is a need for comprehensive national-level research to determine the prevalence and impact of antimicrobial underdosage on individual health, AMR development and healthcare costs—especially in the private sector where PPMVs work. These data are crucial for informed policy interventions and resource allocation.

### Recommendation 4

It is also important to conduct qualitative research to understand patient motivations. Such research would explore patients’ financial constraints, knowledge gaps and decision-making processes to tailor interventions that address their specific needs and challenges.
